# Citrate functionalized Mn_3_O_4_ in nanotherapy of hepatic fibrosis by oral administration

**DOI:** 10.4155/fsoa-2016-0029

**Published:** 2016-10-06

**Authors:** Aniruddha Adhikari, Nabarun Polley, Soumendra Darbar, Damayanti Bagchi, Samir Kumar Pal

**Affiliations:** 1 Department of Chemical, Biological & Macromolecular Sciences, S. N. Bose National Centre for Basic Sciences, Block JD, Sector III, Salt Lake, Kolkata 700106, India; 2 Research & Development Division, Dey's Medical Stores (Mfg.) Ltd., 62, Bondel Road, Ballygunge, Kolkata 700019, India

**Keywords:** fibrosis, hepatoprotective, nanomedicine, nanotherapy, oral administration of drug

## Abstract

**Aim:**

To test the potential of orally administered citrate functionalized Mn_3_O_4_ nanoparticles (C-Mn_3_O_4_ NPs) as a therapeutic agent against hepatic fibrosis and associated chronic liver diseases.

**Materials & methods:**

C-Mn_3_O_4_ NPs were synthesized and the pH dependent antioxidant mechanism was characterized by *in vitro* studies. CCl_4_ intoxicated mice were orally treated with C-Mn_3_O_4_ NPs to test its *in vivo* antioxidant and antifibrotic ability.

**Results:**

We demonstrated ultrahigh efficacy of the C-Mn_3_O_4_ NPs in treatment of chronic liver diseases such as hepatic fibrosis and cirrhosis in mice compared with conventional medicine silymarin without any toxicological implications.

**Conclusion:**

These findings may pave the way for practical clinical use of the NPs as safe medication of chronic liver diseases associated with fibrosis and cirrhosis in human subjects.

## Lay abstract

Hepatic fibrosis is a common response to chronic liver injury from a number of causes including alcohol, toxin, and persistent viral and helminthic infections, which may ultimately lead to hepatic carcinoma. Although billions of people are affected throughout the world, there is no drug available for treatment of this chronic disease. Here, in a preclinical study, we have shown that oral administration of citrate functionalized Mn_3_O_4_ nanoparticles can effectively reduce the extent of liver fibrosis in mice. We have also predicted the underlying therapeutic mechanism that involves mitochondria and antioxidant systems of the body.

**Figure 1.**
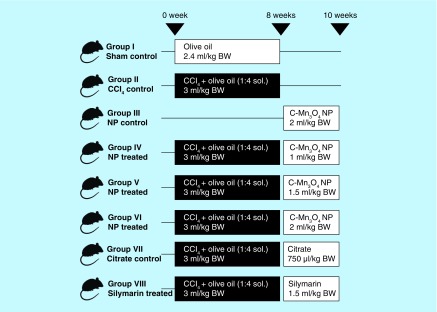
**Group division and treatment protocol of *in vivo* animal studies.** Aqueous solution of NP: OD_430_ = 0.5; Silymarin concentration: 70 mg/ml.

**Figure 2.**
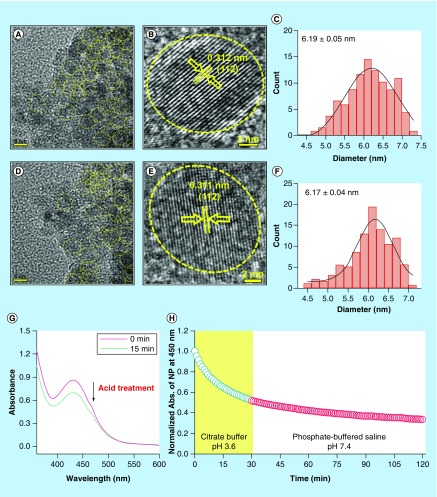
**Effect of acid treatment on physicochemical characteristics of C-Mn_3_O_4_ nanoparticles.** **(A)** TEM image of nanoparticle (NP) at pH 7.4. **(B)** HRTEM image of NP at pH 7.4. **(C)** Size distribution of NPs at pH 7.4. **(D)** TEM image of acid treated (pH 3.6) NP. **(E)** HRTEM image of acid treated (pH 3.6) NP. **(F)** Size distribution of acid treated (pH 3.6) NPs. **(G)** Change in absorbance of NPs due to acid treatment. **(H)** Change in absorbance at 430 nm of NPs during acid treatment and stability after acid treatment. HRTEM: High-resolution TEM; TEM: Transmission electron microscopy.

**Figure 3.**
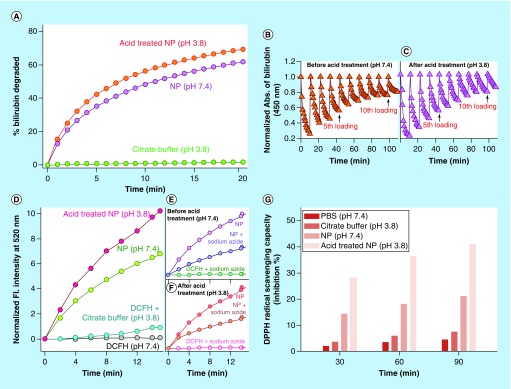
**Physico-chemical characteristics of C-Mn_3_O_4_ nanoparticles.** **(A)** Change in percentage of bilirubin degradation by nanoparticles due to acid treatment. **(B & C)** Recyclability of the catalyst before and after acid treatment, respectively. **(D)** Comparative representation of reactive oxygen species generation capability of nanoparticles due to acid treatment. **(E & F)** Change in reactive oxygen species generation ability due to acid treatment in presence of sodium azide. **(G)** Percentage antioxidant activity as measured by DPPH• assay.

**Figure 4.**
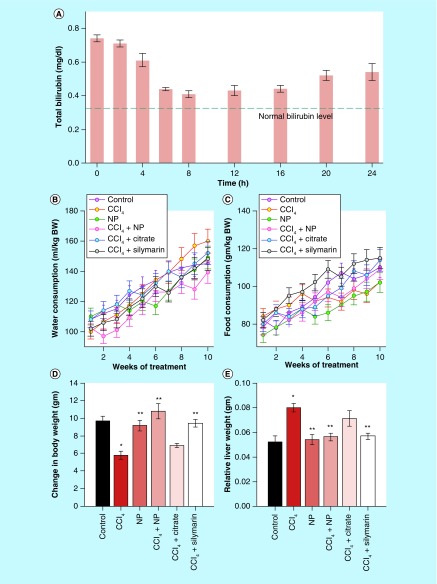
**Effect of nanoparticles on various physiological parameters.** **(A)** Effect on total bilirubin level. Single dose of nanoparticles was able to restore serum bilirubin to almost normal level up to 12 h from hyperbilirubinemia condition. Then it again starts to increase, indicating need for twice a day administration of nanoparticles. **(B & C)** Daily intake of water and food, respectively. **(D)** Change in body weight throughout the experimental period. **(E)** Relative liver weight (liver weight/body weight) after sacrifice. *Values differ significantly from sham control group (Group I) (*p < 0.001). **Values differ significantly from CCl4-treated group (Group II) (**p < 0.05).

**Figure 5.**
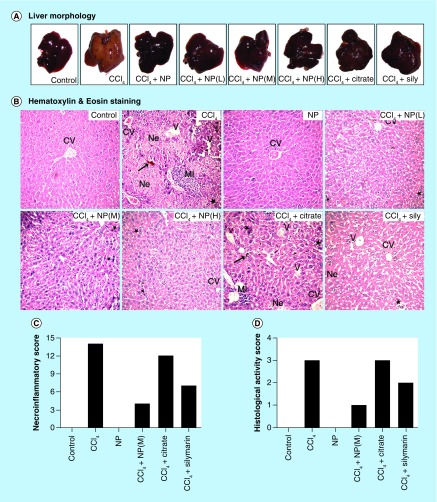
**Effect of nanoparticles on hepatic morphological analysis in CCl_4_-intoxicated mice.** **(A)** Representative photographs of liver after the experimental period. **(B)** Hematoxylin and eosin stained liver sections under microscope of Group I–VIII. **(C & D)** Ishak modified hepatic activity index and METAVIR scoring for necro-inflammatory staging, respectively. Maximal score possible for Ishak HAI is 16, and for METAVIR is 3. *: Increased mitotic activity; →: Hemorrhage; CV: Central vein; HAI: Hepatic activity index; MI: Mononuclear infiltration; Ne: Necrosis, V: Vacuolation.

**Figure 6.**
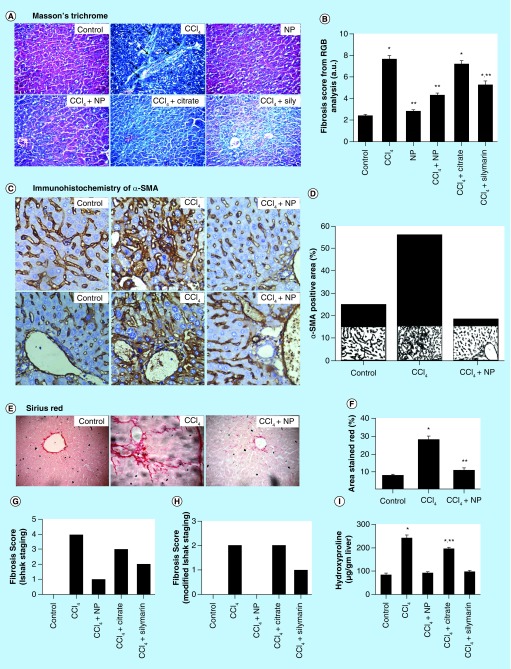
**Effect of nanoparticles on CCl_4_-induced hepatic fibrosis.** **(A)** Masson's trichrome stained liver sections under microscope. CCl_4_-treated group shows marked fibrous septa (portal to potal bridging) however other groups except Citrate show no signs of fibrosis. **(B)** Fibrosis score of different groups as calculated by RGB analysis from MT staining. **(C)** α-SMA immunohistochemistry. Portal arears showed high immunoreactivity in case of CCl_4_ treated mice. **(D)** α-SMA positive area as quantified with ImageJ. **(E)** Sirius red stained liver sections. CCl_4_ treated group shows fibrous expansion of portal areas with portal to portal and occasional portal to central bridging. **(F)** Red stained collagen content as calculated by ImageJ. **(G & H)** Ishak and modified Ishak fibrosis scoring. Highest possible scores are 6 and 4, respectively. **(I)** Hepatic hydroxyproline content. *Values differ significantly from sham control group (Group I) (*p < 0.001). **Values differ significantly from CCl_4_-treated group (Group II) (**p < 0.05).

**Figure 7.**
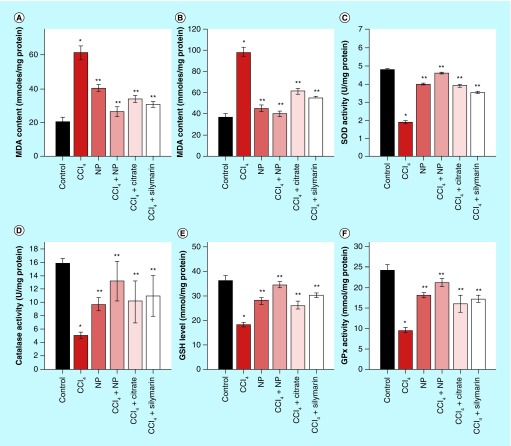
**Effects of orally treated C-Mn_3_O_4_ nanoparticles on liver SOD, catalase, GPx, GSH and MDA content in CCl_4_ intoxicated mice.** **(A)** Serum MDA content. **(B)** MDA content from liver homogenate. **(C)** SOD activity. **(D)** Catalase activity. **(E)** GSH level. **(F)** GPx activity. *Values differ significantly from sham control group (Group I) (*p < 0.001). **Values differ significantly from CCl_4_-treated group (Group II) (**p < 0.05).

**Figure 8.**
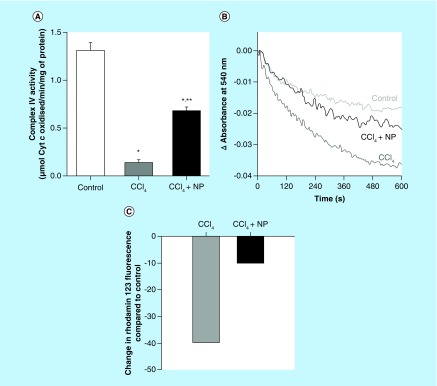
**Effect of C-Mn_3_O_4_ nanoparticles on mitochondria.** **(A)** Complex IV activity. **(B)** Effect on mitochondria permeability transition, measured as decrease in absorbance at 50 nm. **(C)** Change in mitochondrial membrane potential. In CCl_4-_treated mice fluorescence of rhodamine 123 was recovered by 40% compared with control, indicative of decrease in mitochondrial membrane potential (ΔΨ_m_). Treatment with C-Mn_3_O_4_ nanoparticles partially restored the potential. *Values differ significantly from sham control group (Group I) (*p < 0.001). **Values differ significantly from CCl_4_-treated group (Group II) (**p < 0.05).

**Figure 9.**
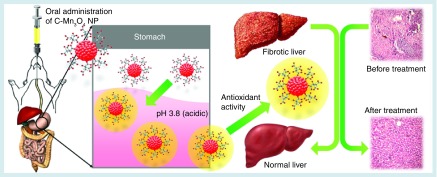
**Schematic representation of the nano-therapy using C-Mn_3_O_4_ nanoparticles.** Oral administration of C-Mn_3_O_4_ nanoparticles recovers liver from severe fibrotic damage caused by chronic CCl_4_ administration. Acidic condition in stomach increases the antioxidant activity of C-Mn_3_O_4_ nanoparticle which in turn reduces the oxidative stress and subsequent recovery of liver takes place.

Manganese oxide nanoparticles (NPs) are now receiving enormous attention due to their unique catalytic activity and associated optical, magnetic, thermal and electrical properties [1]. The many oxidation states of manganese (II, III, IV and VII) provide manganese oxide with significant advantages as a redox medium for scavenging of reactive oxygen species (ROS) [2], which is solely responsible for generating oxidative stress in living system. In vertebrates, liver is the primary target organ for oxidative stress and related damage due to its unique metabolic function and relationship to the gastrointestinal (GI) tract [3]. Despite significant scientific advancement in the field of hepatology in recent years, liver problems are on the rise and account for a high death rate [4–7]. According to the Office for National statistics in the United Kingdom, liver disease is the fifth most common cause of death after heart disease, stroke, chest disease and cancer [8]. Hepatitis is one of the most common liver diseases and the potential causes include autoimmunity, infections from hepatitis viruses, bacteria or parasites, liver injury from alcohol, poisons or hepatotoxic drugs [9]. Chronic hepatitis leads to the recruitment of inflammatory cells, cytokines production and ROS generation which appear to have a central role in development of steatosis and fibrosis [10–12]. Development of fibrosis, particularly cirrhosis, is associated with significant morbidity and mortality [13,14]. Although numerous pharmaceutical agents have been tried, they all lead to unacceptable side effects and limited efficacy during long term therapy [9,15–17]. Therefore, it is necessary and of considerable interest to develop new medicines for treatment of chronic liver diseases. In this context, use of an effective antioxidant without side effects could be used to reduce the oxidative stress that can subsequently lead to the healing of liver insults.

Currently, there is a great deal of interest in the health benefits of inorganic NPs. In the past two decades, several NP-based therapeutics have been successfully introduced for the treatment of cancer, pain and infectious diseases. However, uses of inorganic NPs in treatment of chronic diseases are sparse in literature. One of the major problems in application of nanomedicine against chronic diseases is the route of administration [18]. Oral administrations of drugs are mostly preferred for these types of diseases due to their convenience and compliance. Unfortunately, the NPs are not sufficiently effective because of their nonspecific distribution to the entire body, metabolism in the GI tract, low retention in the lesion area and undesired adverse effects [19]. Citrate functionalized Mn_3_O_4_ NP (C-Mn_3_O_4_ NP) is an inorganic nanoparticle that has previously shown therapeutic promise in safe and symptomatic treatment of hyperbilirubinemia in preclinical models [20].

In the present study, we have demonstrated the potential of orally administered C-Mn_3_O_4_ NPs in effective treatment of severe liver damage in CCl_4_-induced mice model of hepatic fibrosis. To the best of our knowledge, this is the first study that demonstrates direct oral treatment of an inorganic NP (i.e., C-Mn_3_O_4_ NP) without any delivery system can efficiently reduce chronic hepatotoxicity and liver fibrosis through its pH dependent antioxidant activity.

## Experimental section

### Materials

Ethanol amine, 2′,7′-dichlorofluorescin diacetate (DCFH-DA), HCl, H_2_SO_4_, H_2_O_2_ and glycerol were obtained from Merck (NJ, USA). All other chemicals were purchased from Sigma-Aldrich (MO, USA). Millipore water was used whenever required as aqueous solvent. All the chemicals used for this study were of analytical grade and used without further purification.

### Synthesis of C-Mn_3_O_4_ NPs

A reported procedure was followed for template or surfactant-free synthesis of bulk Mn_3_O_4_ NPs at normal temperature and pressure [1,21]. For surface functionalization with citrate, an earlier reported technique was used [1,20]. In brief, as prepared Mn_3_O_4_ NP_s_ were added to 0.5 M aqueous ligand (citrate) solution of pH 7.0 (∼20 mg Mn_3_O_4_ NPs/ml ligand solution) and extensively mixed for 12 h in a cyclomixer. A syringe filter of 0.22 μm diameter was used to eliminate the nonfunctionalized bigger-sized NPs.

### Preparation of acid treated NPs

To mimic the acidic condition of stomach, C-Mn_3_O_4_ NPs were treated with 0.1 M sodium citrate buffer (pH 3.6) and kept for 30 min. After centrifugation, the precipitated NPs were transferred to 0.01 M phosphate-buffered saline (pH 7.4) for further studies (1:10 w/v).

### Characterization techniques

Transmission electron microscopy (TEM) and High-resolution TEM (HRTEM) images were obtained using an FEI TecnaiTF-20 field emission HRTEM operating at 200 kV. Samples were prepared by dropcasting of NP solution (both normal and acid treated) on 300-mesh amorphous carbon-coated copper grid and allowed to dry overnight at room temperature. Absorbance spectra were recorded to inspect the effect of acid treatment on spectral properties and concentrations of the NPs. To compare recyclability of the acid treated NPs to normal ones, we spectrophotometrically monitored bilirubin decomposition kinetics up to ten cycles. The experiment was started with equimolar concentration (10 µM) of bilirubin and catalyst for the first cycle and after every 15 min we added same dose of bilirubin into the reaction mixture. All absorption studies were performed using quartz cuvettes of either 0.4 cm (for serum samples) or 1 cm path length using a Shimadzu Model UV-2600 spectrophotometer.

### ROS generation & free radical scavenging activity of C-Mn_3_O_4_ NPs

*In vitro* ROS generation ability of the NPs and acid treated NPs were evaluated using DCFH-DA following a reported method without any modification [22]. Jobin Yvon Model Fluoromax-3 was used to measure the emission intensity. Free radical scavenging activity of NPs and acid treated NPs were determined using the DPPH assay reported earlier [23]. The capability to scavenge the DPPH• free radical was calculated using the following equation:Equation 1
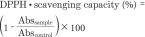



### Animals

Healthy Swiss albino mice of either sex (5–7 weeks old, weighing 27 ± 4 g) were used in this study. Animals were housed in standard, clean polypropylene cages and maintained in controlled laboratory environment (temperature 22 ± 3°C; relative humidity 45–60%; 12 h light/dark cycle). Water and standard laboratory pellet diet for mice (Hindustan Lever, Kolkata, India) were available *ad libitum* throughout the experimental period. All mice were allowed to acclimatize for 1 week prior to experimentation. All animals received human care according to the criteria outlined by the Committee for the Purpose of Control and Supervision of Experiments on Animals, New Delhi, India, and the study was approved by the Institutional Animal Ethics Committee (approval number: Dey's/IAEC/PHA/14/15, dated 31 January 2015).

### Acute toxicity study

Single-dose oral toxicity study was conducted to determine the possible acute toxicity of C-Mn_3_O_4_ NPs following the general principles of the OECD guideline 423 [24] with some adjustments. Twelve female mice were divided into four groups: one control group (received 0.2 ml MilliQ water) and three experimental groups (received either 500, 2000 or 5000 mg/kg body weight [BW] of NPs). All the animals were kept in fasting condition overnight prior to feeding. Behavior, mortality and BW were monitored daily for a period of 14 days.

### *In vivo* catalytic activity of NPs

To investigate whether the NPs are active in *in vivo* system even after oral administration, serum bilirubin concentration was monitored in CCl_4_-induced mice model of hyperbilirubinemia. Bilirubin level was increased in a group of 14 mice through intraperitoneal injection of CCl_4_ (25% CCl_4_ in olive oil, 3 ml/kg BW in alternative days for 8 weeks). After single oral feeding of NPs (1.5 ml of OD_430_ 0.5/kg BW), serum bilirubin concentration was measured in every 2 h up to 24 h to evaluate the catalytic efficiency.

### *In vivo* distribution of NPs

The manganese contents in the liver (24 h after treatment) and blood (2 h after treatment) were estimated using inductively coupled plasma atomic emission spectroscopy (ICP-AES; ARCOS, Simultaneous ICP Spectrometer, SPECTRO Analytical Instruments GmbH, Germany) at SAIF, IIT Bombay, India. The samples were prepared using open acid digestion method. In brief, dried tissues were dissolved in HNO_3_ (15 ml), H_2_SO_4_ (10 ml) and H_2_O_2_ (5 ml), heated at 120°C until only a residue remained and then diluted with deionized water to 10 ml.

### Treatment protocol

The animals were randomized into eight groups (n = 10 in each group). The division of groups and treatment protocol is described in Figure 1. Intraperitoneal injection of CCl_4_ solution was used to introduce hepatic fibrosis and chronic hepatotoxicity. NPs were administered as aqueous solutions. Standard hepatoprotective drug Silymarin was used as control. All treatments were done via oral administration. At the end of the experiment, the animals were kept in fasting condition overnight and sacrificed by cervical dislocation.

### Histopathological examination

After collection of blood, liver was excised, washed with ice-cold phosphate buffer and dried with tissue paper. It was weighed and fixed in neutral formalin solution (10%), dehydrated in graduated ethanol (50–100%), cleared in xylene and embedded in paraffin, and 4–5 µm thick sections were cut, deparaffinized, hydrated and stained with hematoxylin and eosin (H/E). Masson's trichrome (MT) and Sirius red (SR) staining were also performed to quantify the extent of fibrotic damage. Histopathological changes were examined under the microscope (Olympus BX51). Fibrosis score was calculated from RGB (Red-Green-Blue) image analysis performed using Matlab^®^ R2014b, MathWorks, Inc. (MA, USA) using the formula:Equation 2
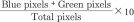



### Immunohistochemistry

Paraffin-fixed liver tissue slices were sectioned, deparaffinized, rehydrated and immersed in 3% H_2_O_2_ for 10 min to block endogenous peroxidase activity. Antigen retrieval was performed in citrate buffer (pH 6.0) in a microwave oven for 15 min. Bovine serum albumin (5%) was used to block nonspecific protein binding. The sections were incubated with α-smooth muscle actin (α-SMA) primary antibody overnight at 4°C. The sections were subsequently washed with phosphate-buffered saline and incubated with horseradish peroxidase-conjugated goat antimouse IgG secondary antibodies, followed by incubation for 5–10 min with 3,3′-diaminobenzidine tetrachloride. Stained slides were analyzed using high-power field images captured under microscope (magnification × 400; Olympus BX51). Computer-assisted semi-quantitative analysis was used to evaluate the α-SMA positive areas using ImageJ software following reported literature [25]. The data for α-SMA staining were expressed as the mean percentage of the positively stained area over the total tissue section area.

### Scoring of fibrosis

Scoring of fibrosis was done by an independent pathologist unaware of the experiment using random microscopic field images of H/E, MT, SR and immunohistochemically stained liver sections. For the scoring of hepatic necrosis we used METAVIR system as well as Ishak Modified Hepatic Activity Index. Fibrosis score was calculated following both original and modified Ishak Staging.

### Hepatic hydroxyproline measurement

Hepatic hydroxyproline content was measured using the method described elsewhere [26]. In brief, snap-frozen liver specimens (200 mg) were weighed, hydrolyzed in 6 M HCl overnight at 100°C (purified 4-hydroxy-L-proline standards for 20 min at 120°C). Free hydroxylproline content from each hydrolysate was oxidized with Chloramine-T. The addition of Ehrlich reagent resulted in the formation of a chromophore whose absorbance was read at 550 nm. Data were normalized to liver wet weight.

### Serum isolation

For biochemical studies, blood samples were collected in sterile tubes (nonheparinized) from retro-orbital plexus just before sacrifice and allowed to clot for 45 min. Serum was separated by centrifugation at 600 × g for 15 min.

### Measurement of liver function enzymes

All serum samples were sterile, hemolysis-free, and were kept at -20°C before determination of the biochemical parameters. The activities of alanine aminotransferase (ALT), aspartate aminotransferase (AST), γ-glutamyltransferase (GGT), alkaline phosphatase (ALP), total bilirubin, direct bilirubin and total protein in plasma were determined using commercially available test kits (Autospan Liquid Gold, Span Diagnostics Ltd., Gujarat, India) following the protocols described by the corresponding manufacturers.

### Liver homogenate preparation

Samples of liver tissue were collected, homogenized in cold 0.1 mM phosphate buffer (pH 7.4), and centrifuged at 10,000 r.p.m. at 4°C for 15 min. The supernatants were collected to determine the activity of SOD, CAT, GPx and GSH as well as the content of malondialdehyde (MDA).

### Assessment of lipid peroxidation & hepatic antioxidant status

The supernatants were used to determine the activity of SOD, CAT, GPx and GSH as well as the content of MDA. Degree of lipid peroxidation was determined in terms of thiobarbituric acid reactive substances (TBARS) formation using a reported procedure [27]. SOD and CAT activities were estimated following methods described by Kakkar *et al*. [28] and Britton and Mehley [29,30], respectively. Hepatic GSH level was determined by the method of Ellman with slight modification [31,32].

### Hematological study

For hematological studies, smears were drawn from heparinized blood and Sysmax-K1000 Cell Counter was used for blood cell count. Studied parameters included hemoglobin, total red blood cell, reticulocyte, hematocrit, mean corpuscular volume, mean corpuscular hemoglobin, mean corpuscular hemoglobin concentration, platelets and total white blood cell.

### Mitochondria isolation

Mitochondria were isolated from mouse livers according to the method of Graham [33] with some slight modifications. In brief, livers were excised and homogenized in liver homogenization medium containing 225 mM D-mannitol, 75 mM sucrose, 0.05 mM EDTA, 10 mM KCl, 10 mM HEPES (pH 7.4). The homogenates were centrifuged at 600 × *g* for 15 min and resulting supernatants were centrifuged at 8500 × *g* for 10 min. The pellets were washed thrice and resuspended in same buffer. All procedures were done at 4°C. Protein concentration was determined using commercially available kit (Autospan Liquid Gold, Span Diagnostics Ltd., India) following the protocol described by the manufacturer.

### Complex IV (of respiratory chain) activity

Total complex IV activity was measured spectrophotometrically using isolated mitochondria [34]. Briefly, reduced cytochrome c was prepared by mixing cytochrome c and ascorbic acid in potassium phosphate buffer. Complex IV activity was taken as the rate of ferrocytochrome c oxidation to ferricytochrome c, detected as the decrease in absorbance at 550 nm.

### Measurement of mitochondrial membrane permeability transition

Opening of the pore causes mitochondrial swelling, which results in reduction of absorbance at 540 nm. Mitochondrial permeability transition (swelling assay) was monitored as changes at 540 nm at 10 s intervals over 10 min time with 250 µg mitochondrial protein in the swelling buffer, which contained 120 mM KCl (pH 7.4) and 5 mM KH_2_PO_4_.

### Measurement of mitochondrial membrane potential

The mitochondrial membrane potential (ΔΨ_m_) was measured using the fluorescent probe rhodamine 123 (Sigma) [35,36]. Because rhodamine 123 is a cationic dye, it accumulates in the mitochondria driven by ΔΨ_m_. Under appropriate loading conditions, the concentration of rhodamine 123 within the mitochondria reaches sufficiently high levels that it quenches its own fluorescence (λ_ex_ = 503 nm, λ_em_ = 527 nm). If the mitochondria depolarize, rhodamine 123 leaks out into the cytoplasm and is associated with a reduction in the amount of quenching. Thus the changes in ΔΨ_m_ are revealed as changes in total fluorescence intensity following the method of Chen [37].

### Statistical analysis

All quantitative data are expressed as mean ± SD unless otherwise stated. One-way analysis of variance followed by Tukey's multiple comparison test was executed for comparison of different parameters between the groups using a computer program GraphPad Prism (version 5.00 for Windows), GraphPad Software (CA, USA). p < 0.05 was considered significant.

## Results & discussion

The present study was conducted to explore the potential of C-Mn_3_O_4_ NPs as orally administered drug against chronic liver diseases. Liver, being the major detoxifying organ, receives 75% of the blood directly from gastrointestinal viscera and spleen [3]. All orally applied drugs need to pass through the highly acidic stomach before entering the hepatic circulation and it is well known that pH and ionic conditions greatly affect stability and functionalization of NPs [38,39]. Therefore, the effect of pH (mimicking the stomach condition) on physicochemical characteristics and activity of NPs was evaluated. The C-Mn_3_O_4_ NPs applied in this study shaped nearly spherical (Figure 2A & B), with size distribution of about 6–10 nm (mean particle size: 6.19 ± 0.05 nm) (Figure 2C). HRTEM image of single NP (Figure 2B) confirmed the crystalline nature of it with interfringe distance of 0.312 nm (corresponding to the (112) planes of the Mn_3_O_4_ tetragonal crystal lattice). Upon acid treatment there was no significant change in shape, size (mean particle size: 6.17 ± 0.04 nm) or crystallinity (interfringe distance of 0.311 nm) of the NPs as evident from Figure 2D–F. However, the effective concentration of NPs in solution decreased during acid treatment which is clear from time dependent decrease in absorption peak of NPs at 430 nm (resembles d–d transition of Mn) (Figure 2G). Transfer of acid treated NPs to neutral pH showed little or no change in concentration over time, indicating its stability after acid digestion in stomach (Figure 2H).

The catalytic efficacy of C-Mn_3_O_4_ NPs to degrade bilirubin in dark condition [40] was monitored to compare the activities of neutral and acid treated NPs. The bilirubin degradation kinetics (Figure 3A) clearly showed an increase in bilirubin degradation activity of NPs upon acid treatment. The increased catalytic activity caused by acid treatment is consistent with the fact that, at higher pH, Mn^3+^ in the NPs surface is stable due to comproportionation of Mn^2+^ and Mn^4+^ and does not tend to react with bilirubin [40] whereas in acidic pH, Mn^3+^ ions are unstable and tend to disproportionate into Mn^2+^ and Mn^4+^ which are highly reactive toward bilirubin [41]. The recyclability of catalyst was also tested. Figure 3B and C describes that both neutral and acid treated NPs could be recycled up to ten cycles.

In various studies, it has been observed that inorganic NPs have a tendency to produce ROS in solution, and C-Mn_3_O_4_ NPs are no exemption to this. Nonfluorescent DCFH-DA is a useful indicator of ROS, which is oxidized to fluorescent DCF in presence of ROS. The emission intensity at 520 was monitored with time to evaluate the extent of ROS generation. We observed an increase in ROS generation upon to acid treatment (Figure 3D). The nature of ROS was found to be singlet oxygen, because emission of DCF reduced significantly in presence of sodium azide, a well-known singlet oxygen quencher (Figure 3E & F).

It is also well known that the hepatoprotective effects of a compound largely depend on its antioxidant capacity. So, we evaluated the antioxidant capacity of C-Mn_3_O_4_ NPs (both neutral and acid treated) using DPPH• method, a nonenzymatic test widely used to provide basic information on the free radical scavenging ability of compounds. Figure 3G clearly indicates that C-Mn_3_O_4_ NPs provide substantial radical scavenging activity and can act as an antioxidant. Moreover, acid treatment significantly increased its free radical scavenging capability (Figure 3G). This antioxidant activity of C-Mn_3_O_4_ NPs is likely to involve redox reaction between the Mn(II) and Mn(III) states due to ligand to metal charge transfer (originated from the interaction of Mn^3+/4+^ centers in the NPs with the surface bound citrate ligands). The formation of complex between Mn and an anion causes a decrease in the redox potential of the Mn (II) ←→Mn(III) couple, enhancing the disproportionation of Mn(III) to Mn(II) [42]. Previous studies have revealed that Mn(II) can act as a free radical scavenger [43]:Equation 3
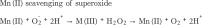

Equation 4
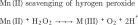



In the previous section we have discussed that acid treatment increased effective concentration of Mn(II) state in NP surface, in turn facilitating the free radical scavenging reactions indicated in Equations 3 & 4.

For assessing the maximal-tolerated dose of C-Mn_3_O_4_ NPs, we executed single-dose acute toxicity study following OECD guideline. Oral administration of C-Mn_3_O_4_ NPs did not cause any mortality throughout the experimental period for all three dose groups. During the study period no behavioral and physical symptoms of acute toxicity such as decreased activity or decreased uptake of food and water were observed.

In order to investigate the catalytic effectiveness of the NPs *in vivo*, serum bilirubin concentration was monitored in a time dependent manner after single oral administration of the NPs in hyperbilirubinemia mice model. The results (as described in Figure 4A) indicated that the catalytic efficiency of the NPs was retained for almost 12 h in circulatory system, after that it started to diminish resulting in consequent rise in the bilirubin concentration. The decreased activity may be attributed to excretion of the NPs from the body.

The internalization of NPs from GI tract is a delicate subject that should be addressed carefully. We estimated Mn content in liver and circulation by ICP-AES, in order to pursue an idea about internalization and biodistribution of NPs. The results show increased deposition of Mn in liver 12 h after treatment with C-Mn_3_O_4_ NPs (4.04 ± 0.2 µg/gm tissue compared with 2.54 ± 0.1 µg/gm tissue of control; p < 0.05). The Mn content of blood also increased from 0.81 ± 0.1 µg/ml to 1.54 ± 0.3 µg/ml (p < 0.05) after 2 h of treatment.

Figure 5B shows the change in BW of mice during the experimental period of 10 weeks. Growth of mice was significantly retarded upon CCl_4_ injection (Group II). Three weeks administration of C-Mn_3_O_4_ NPs and Silymarin improved the growth of CCl_4_ intoxicated mice almost comparable to the normal ones (Group I); however, C-Mn_3_O_4_ NPs exhibited slightly better result than Silymarin. Figure 4B & C shows consumption of water and food, respectively. Significant decrease in food uptake and an increase in water uptake for the CCl_4_ intoxicated group signifies the toxicity induced by the xenobiotics. Figure 4D shows the change in BW of the mice during experimental period. Increase in relative liver weight was observed in CCl_4_ treated mice (Figure 4E) which may be due to enlargement of liver as well as accumulation of lipids, in other words, triglycerides. C-Mn_3_O_4_ and Silymarin both seems to decrease the fat deposition effectively.

CCl_4_ is a well-known hepatotoxic agent widely used to study hepatoprotective activity of new drugs in *in vivo* experimental models of liver cirrhosis and fibrosis [44–46]. Chronic CCl_4_ administration induces critical liver damage in mice which in turn simulates a condition of acute hepatitis showing similar symptoms as humans [45,47–48]. The liver fibrosis induced by CCl_4_ is the result of reductive dehalogenation. The highly reactive metabolite trichloromethyl radical (•CCl_3_) is formed from the metabolic conversion of CCl_4_ by cytochrome P-450. These radicals readily interact with O_2_ to form a more reactive trichloromethylperoxy radical (CCl_3_OO•) [49], which is capable of binding to protein or lipid, or of abstracting hydrogen atoms to form chloroform, which leads to lipid peroxidation and liver damage as well as plays significant role in liver pathogenesis [50–52].

In order to assess the protective effect of C-Mn_3_O_4_ NPs against CCl_4_-induced chronic hepatitis, structural changes of H/E stained liver sections were analyzed under microscope. Figure 5A shows the morphometric condition of the liver throughout experimental groups. Effect of CCl_4_ toxicity was evident in case of Group II and VII. Liver sections of the vehicle control animals stained with H/E showed a typical hepatic architecture with hepatic plates directed from the portal triads toward the central vein where they freely anastomose. Irregularly dilated normal sinusoids and spaces of Disse accompanied by healthy hepatic cells with well-preserved cytoplasm and prominent nucleus have been seen in this group of mice (Figure 5B). In the CCl_4_-intoxicated mice (Group II; Figure 5B), moderate to severe hepatocellular vacuolation along with massive centrilobular necrosis and hydropic degeneration was detected. Increased cellular mitosis as well as dilation of Disse spaces with focal disruption of the sinusoidal endotheliam, inflammatory infiltrations into the portal triads and distortion of CVs have also been observed. Occurrence of mononuclear cell infiltrations, hemorrhage and fatty degeneration is in agreement with previous studies and further confirms acute liver injury caused by CCl_4_. The animals treated with NPs (Group IV–VI) and Silymarin (Group VIII) revealed slight to mild hepatocellular vacuolation and better preservation of the normal liver architecture (Figure 5B). All of these treated groups displayed occasional periportal inflammatory infiltrate, smaller dilation of Disse space and renovation of compact liver structure. Although treatment with both NPs and Silymarin reversed the downgradation in hepatic architecture, NPs showed better activity in respect to Silymarin as observed from hepatic morphological analysis. The nontoxic effects of NPs on hepatocytes were again confirmed as the animals treated with only C-Mn_3_O_4_ NPs (Group III; Figure 5B) showed normal liver architecture comparable to vehicle control group. Citrate has shown no or very little restorative effect on hepatic morphology (Group VII; Figure 5B). Based on the microscopic observations, we assessed the necroinflammatory changes of tissue sections using Ishak modified hepatic activity index (Figure 5C) and METAVIER system (Figure 5D) [53,54]. In the Ishak's grading highest score possible is 18 and for METAVIER it is 3. Tissue sections from CCl_4_-induced mice scored 14 and 3 (severe), respectively. However treatment with C-Mn_3_O_4_ NPs decreased it to level of the control (0 for both scoring). So, according to the microscopic examinations, severe cellular liver damage induced by CCl_4_ was remarkably reduced by oral administration of the NPs.

For evaluation of fibrosis and its recovery, we used three staining methods: Masson's trichrome, Sirius red and immunohistochemical staining of α-SMA. Masson's trichrome staining is a well-established technique to demonstrate the accumulation of collagen fibers in the liver tissue during hepatic fibrosis and cirrhosis [55]. The results of the Masson's trichrome staining demonstrating accumulation of matured collagen fibers (stained blue) during CCl_4_-induced hepatic fibrosis and also the role of C-Mn_3_O_4_ NPs to prevent collagen synthesis and deposition in the liver are depicted through Figure 6A. Trichrome staining of normal liver did not show any collagen deposition (Figure 6A; control), whereas those from CCl_4_-induced mice showed bile duct proliferation with dense fibrous septa with portal to portal bridging (Figure 6A; CCl_4_) and increased deposition of collagen fibers around the congested central vein, indicating fibrosis. However the liver sections from CCl_4_-induced fibrotic mice administered with NPs had fewer fibers (Figure 6A; CCl_4_+NP), while those treated with Citrate and Silymarin had more fibers than NP-treated ones (Figure 6A; Sily). There was no fibrosis and deposition of blue collagen fibers in case of NP control group (Figure 6A; NP). The numbers of blue pixels relative to the total pixels in Masson-stained liver sections were measured to quantify collagen fibers. CCl_4_ significantly (p < 0.001) increased the number of blue pixels in liver sections (Figure 6B). Administration with NPs resulted in significant lower number of blue pixels, but Silymarin-treated ones had a significant (p < 0.05) higher number of blue pixels compared with both control and NP-treated ones. Thus, direct evaluation of extra cellular matrix (ECM) deposition in hepatic tissue by Masson's trichrome staining clearly depicts the therapeutic efficiency of NPs against chronic hepatic fibrosis.

Hepatic stellate cell (HSC) activation plays a key role in liver fibrosis at the early phase and activated HSC is accompanied with high expressions α-SMA proteins. So, hepatic α-SMA immunoreactivity, which detects activated HSC, a definitive marker of fibrotic liver, has been shown in Figure 6C. With regard to the distribution of α-SMA-positive fibrogenic cells, in the livers of control animals, α-SMA immunopositivity was restricted to the smooth musculature belonging to the arterial tunica media, as well as to the wall of majority of portal and central veins, while other liver cells remain negative (Figure 6C; Control). CCl_4_ strongly induced perisinusoidal α-SMA expression, which was recognized as activated HSCs, through affected lobule, connected between themselves with thin, ‘bridging’ immunopositivity (Figure 6C; CCl_4_). The livers of mice receiving C-Mn_3_O_4_ NPs showed staining pattern similar to control animal (Figure 6C; CCl_4_+NP) with sporadic α-SMA positivity. The α-SMA positive area was calculated and shown in Figure 6D. It clearly showed that CCl_4_ treatment caused more than twofold increase in α-SMA level, which upon treatment with NPs decreased to a level comparable to control animals, indicating an attenuation of the fibrogenic properties of HSCs after administration of C-Mn_3_O_4_ NPs.

Sirius red selectively stains collagen, the most abundant ECM protein produced during fibrogenesis. Figure 6E shows Sirius red stained liver sections of different groups. CCl_4_ treatment caused fibrous expansion of portal areas with portal to portal bridging, occasional portal to central bridging and characteristic perisinusoidal chicken wire fence pattern, indicative of progression of fibrosis. Treatment with C-Mn_3_O_4_ caused marked decrease in fibrous extensions which has also been reflected in Figure 6F, quantification of the Sirius red stained collagen area.

On the basis of histological findings, we applied scoring to the livers of different groups. Both Ishak and Ishak modified fibrosis staging was performed (Figure 6G & H, respectively). After 8 weeks of CCl_4_ administration, most mice had fibrous portal expansion with short fibrous septa (Ishak 3), and occasionally progressed to complete bridging fibrosis with appearance of a few of regenerative nodules (Ishak 4). However, treatment with C-Mn_3_O_4_ NPs decreased the extent of fibrosis, reducing the score to normal.

The degree of fibrosis was also assessed using the collagen quantitation by measuring hydroxyproline content, a product of collagen metabolism. The results, as depicted in Figure 6G, indicates CCl_4_-induced hepatic fibrosis with almost threefold increase (p < 0.05) in hydroxyproline content. Treatment with NPs decreased that level almost to control, which was also apparent in histological and immunohistochemical findings, further confirming protective effect of C-Mn_3_O_4_ NPs against fibrosis.

Results of histopathological studies are further supported by changes in biochemical parameters in serum. In order to assess the protective effect of C-Mn_3_O_4_ NPs against CCl_4_-induced chronic hepatitis, serum activities of various hepatic lysosomal enzymes were used as diagnostic indicators (Table 1). The dramatically elevated serum levels of transaminases i.e., AST and ALT (∼400 and ∼200%, respectively) after CCl_4_ treatment have been attributed to damaged structural integrity of the liver [31,51]. Leakage of large quantities of these enzymes from liver pool into the blood stream is associated with massive centrilobular necrosis, ballooning degeneration and cellular infiltration of the liver. Other liver specific preclinical and clinical biomarkers showed same trend. Elevated levels of ALP (∼265%), GGT (∼100%), TB (∼330%), DB (∼200%) and decrease in total protein concentration further confirmed chronic hepatitis induced by CCl_4_ [31]. Treatment with C-Mn_3_O_4_ NPs at a dose of 1.5 ml (OD_430_ 0.5)/kg BW for 14 days considerably reduced the elevated serum levels of aforementioned enzymes to almost normal (AST ∼80%, ALT ∼55%, ALP ∼70%, GGT ∼30%, TB ∼84%, DB ∼80% compared with CCl_4_ treated group; p < 0.05) with subsequent improvement in serum protein concentration (45% compared with CCl_4_-treated group; p < 0.05), implying that C-Mn_3_O_4_ NPs tended to prevent damage and suppressed the leakage of enzymes. Treatment with a well-known hepatoprotective drug Silymarin also improved the liver parameters, however with lesser efficacy (AST ∼67%, ALT ∼50%, ALP ∼65%, GGT ∼22%, TB ∼73%, DB ∼72% compared with CCl_4_-treated group; p < 0.05). Moreover, it could not restore the above mentioned enzymes particularly AST and ALT (1.8- and 1.4-times higher, respectively, compared with control; p < 0.05) to normal level within the treatment period compared with NPs. This clearly implies that NPs could heal hepatic damage faster than the conventional drug Silymarin. The liver function parameters for the NP control group (group III) remained almost same as the vehicle treated group (Group I) demonstrating nontoxicity of NPs on liver at administered dose. No significant improvement in the citrate control group confirmed ineffectiveness of ligand citrate alone in prevention of hepatotoxicity.

The ratio of serum activities of AST and ALT (De Ritis Ratio) is useful in differential diagnosis and classification of hepatic disorders. For normal individuals, this ratio varies from 0.7 to 1.4 (as in case of Group I; 1.08) [17]. The value of De Ritis Ratio in case of CCl_4_-administered group (Group II) has increased to 1.85. This increased value of >1.5 along with ALT:ALP ratio of 1.42 (<2.0) is indicative of intrahepatic lesion formation and chronic liver disorders such as fibrosis, post necrotic cirrhosis, drug-induced cholestasis, etc. [16,17]. Treatment with C-Mn_3_O_4_ NPs restored the De Ritis ratio to normal level (Group V; 0.80), whereas conventional drug silymarin (Group VIII) decreased it to 1.18. However other two C-Mn_3_O_4_ NP dose control groups (group IV and VI) also showed similar activities with reduced efficiency.

Rapid lipid peroxidation of the membrane structural lipids has been proposed as the basis of CCl_4_ liver toxicity and a marker of fibrosis. So, we monitored the levels of MDA, an index of oxidative damage and one of the decomposition products of peroxidased polyunsaturated fatty acids, to evaluate the effect of C-Mn_3_O_4_ NPs treatment on CCl_4_-induced liver peroxidation. As shown in Figure 7A & B, significant increase of MDA (∼172 and ∼205%, respectively, for hepatic and serum MDA content; p < 0.05) in the CCl_4_-treated group confirmed that oxidative damage had been induced. Consistent with liver function tests, treatment with C-Mn_3_O_4_ NPs and Silymarin significantly reduced both hepatic (∼59 and ∼44%, respectively; p < 0.05) and serum (∼57 and ∼49%, respectively; p < 0.05) MDA content.

SOD, CAT and GPx comprise the major antioxidant system in mammalian cells, which constitutes a mutually supportive team for defense against ROS [56]. SOD converts superoxide anions to H_2_O_2_, which is further converted to H_2_O with the help of GPx and CAT. SOD also inhibits hydroxyl radical production [9]. Maintaining the balance between ROS and antioxidant enzymes is crucial for prevention of oxidative damage [57] which can damage all single aspects of a cell, including its protein, lipids and DNA [9,58]. As shown in Figure 7C–F, CCl_4_-induced substantial modifications to the hepatic antioxidant enzymes and significantly decreased hepatic SOD (∼60%), CAT (∼68%) and GPx (∼62%) activities. Treatment with orally administered NPs and Silymarin considerably elevated the antioxidant enzyme levels. Citrate also showed some amount of efficacy in reversal of antioxidant defense mechanism. In case of NP control group (Group III), some pro-oxidant effect was observed. This is because of the inherent property of the NPs to produce ROS in solution as described in our *in vitro* studies. However this change has not damaged the liver and it has no effect on liver marker enzymes. GSH, a nonenzymatic antioxidant, plays excellent role in protection of cells from CCl_4_-induced hepatotoxicity [9]. GSH combines with trichloromethyl radical, in presence of GST catalytic activity, which in turn contributes to detoxification of CCl_4_. GSH stores are markedly depleted, especially when liver necrosis initiates. In this study, we observed decrease in hepatic GSH (∼50%) level upon CCl_4_ administration. Treatment with C-Mn_3_O_4_ NPs restored GSH level to the normal ones. The effect could be due either to the *de novo* synthesis of GSH, its regeneration or both. The observed *in vivo* ability of C-Mn_3_O_4_ NPs in protection against lipid peroxidation and oxidative damage may involve various mechanisms. First, redox reaction between the Mn(II) and Mn(III) states in C-Mn_3_O_4_ NPs due to ligand to metal charge transfer may help it to act as a scavenger of hydroxyl and superoxide radicals (details are discussed in previous section of the text). Second, it may act as a chain-breaker in inhibiting iron-induced lipid peroxidation chain reactions [59,60], and as proposed in other studies, Mn(II) may scavenge peroxyl lipid radicals via the following reaction [61,62]:Equation 5
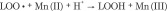



Third, during comproportionation/disproportionation reactions small amounts of Mn^2+^ is dissoluted in solution [41], which being a cofactor can enhance Mn-SOD activity (an essential isozyme of SOD in antioxidant defense system) [63]. Fourth, it can promote the synthesis of metallothionein, which then scavenges oxidant radicals and fifth, being a trivalent cation, Mn(III) can interfere with the effects of Fe^3+^, which is known to be involved in reactive oxidant radical generation.

To elucidate the possible link between antifibrotic and axtioxidative properties of NPs, we further studied its effect on mitochondria. Increasing evidences support the dependency of mitochondrial defense mechanisms on the cytosolic pool of reducing equivalents such as GSH. Depletion of these equivalents (also evident in our study) in the cytosol has direct consequences on the mitochondrial redox state. Previous studies have shown that Complex IV in the respiratory chain plays a critical role in oxidative stress and associated apoptosis [64]. So, at first, we measured the activity of Complex-IV and found them to be significantly decreased in CCl_4_ treated mice (Figure 8A), which is in accordance with previous studies [65,66]. Although, treatment with C-Mn_3_O_4_ NPs has significantly (p < 0.05) increased its activity, normal level was not restored. Ca^2+^-induced liver mitochondria permeability transition is a useful model for evaluating the effects of drugs or other substances on mitochondrial function [67]. Our data clearly show that, the mitochondria isolated from the CCl_4_ intoxicated-group were more sensitive to Ca^2+^ as shown by a quick decline of 540 nm absorbance upon addition of Ca^2+^. C-Mn_3_O_4_ NPs attenuated Ca^2+^-induced mitochondria permeability transition, as shown by slow decline of A_540_ that mimicked the control group (Figure 8B). This indicates a protective role of C-Mn_3_O_4_ NPs on normal MTP of mitochondria. In addition, we investigated the effect of C-Mn_3_O_4_ NPs on mitochondrial membrane potential (MMP). CCl_4_ intoxication caused dissipation of MMP, reflected in less quenching of initial rhodamine 123 fluorescence which was in agreement with previous reports [68]. However, treatment with NPs, prevented the collapse in MMP (Figure 8C).

In order to evaluate any potential toxicity of the NPs, we examined necessary hematological parameters of Group I–III, V and VII–VIII. All the parameters are comparable with sham control and showed no toxicity except the CCl_4_-induced group. The number of white total blood corpuscles in CCl_4_-induced is significantly higher than Group I, since the liver tissues were infiltrated by huge amount of inflammatory cells due to fibrotic damage (also evident from histological studies). Treatment with NPs significantly decreased the inflammatory infiltration. Data are presented in Table 2.

## Conclusion

In conclusion, the present study demonstrated that C-Mn_3_O_4_ NPs, when administered orally, can protect liver from CCl_4_-induced cirrhosis, fibrosis and oxidative stress due to increased antioxidant properties upon acid treatment in stomach (Figure 9 summarizes the whole study). Their possible promising therapeutic role against oxidative stress and related chronic liver diseases deserves consideration. However, cautions must be taken as there is prevalent debate about nanotoxicity. Detailed toxicity study and more preclinical trials are required before they reach the clinics for use in prevention of liver diseases.

## Future perspective

In the years to come, advances in engineering nanomaterials with exquisite size and shape control will expand their use in biomedical applications and open the door of personalized medicine. Our study has shown the potential use of Mn-based NPs directly as a therapeutic agent against chronic liver disease. However, detailed molecular study is required to get further insight into the mechanism of action of the NPs. A detailed toxicological assessment, pharmacokinetic study and experimentation on biodistribution of the NPs will help to confirm the potential of this nanoparticle in preclinical studies more strongly and lead the way to clinical trials.

**Table 1.** **Effect of C-Mn_3_O_4_ nanoparticle on liver function parameters of CCl_4_ intoxicated mice.**

**Group**	**Design of treatment**	**AST (IU/l)**	**ALT (IU/l)**	**ALP (IU/l)**	**GGT (IU/l)**	**Total bilirubin (mg/dl)**	**Direct bilirubin (mg/dl)**	**Total protein (gm/dl)**
I	Sham control	87.3 ± 15.4	80.4 ± 12.1	44.5 ± 5.8	3.1 ± 0.26	0.32 ± 0.04	0.18 ± 0.01	8.84 ± 0.09^§^
II	CCl_4_ control	427.5 ± 62.1^‡,§^	230.1 ± 35.6^‡,§^	161.2 ± 14.3^‡,§^	6.3 ± 0.41^‡,§^	1.28 ± 0.04^‡,§^	0.54 ± 0.02^‡,§^	5.11 ± 0.07^‡,§^
III	NP control	95.6 ± 12.5^†^	88.2 ± 7.3^†^	59.8 ± 4.9^†^	3.8 ± 0.21^†^	0.18 ± 0.05^†^	0.09 ± 0.01^†^	8.12 ± 0.64^†,§^
IV	CCl_4_ + NP (L)	142 ± 12.8^†,‡^	126.8 ± 14.3^†,‡^	95.4 ± 11.1^†,‡^	5.7 ± 0.67^†,‡^	0.24 ± 0.05^†,‡^	0.13 ± 0.01^†,‡^	6.24 ± 0.09^†,‡,§^
V	CCl_4_ + NP (M)	82.7 ± 11.2^†,§^	102.57 ± 5.8^†,§^	50.1 ± 4.5^†,§^	4.4 ± 0.23^†,§^	0.21 ± 0.07^†,§^	0.11 ± 0.01^†,§^	7.44 ± 0.11^†,‡,§^
VI	CCl_4_ + NP (H)	115.4 ± 13.6^†^	108.5 ± 10.2^†^	64.5 ± 6.8^†^	5.2 ± 0.62^†^	0.22 ± 0.04^†^	0.11 ± 0.01^†^	6.84 ± 0.14^†,‡^
VII	CCl_4_ + Citrate	324.6 ± 45.4^†,‡^	194.6 ± 22.7^†,‡^	145.7 ± 12.3^†,‡^	5.9 ± 0.52^†,‡^	0.99 ± 0.06^†,‡^	0.32 ± 0.03^†,‡^	5.70 ± 0.03^‡,§^
VIII	CCl_4_ + Silymarin	137.9 ± 17.8^†,‡^	116.6 ± 14.3^†,‡^	55.6 ± 3.2^†,‡^	4.9 ± 0.51^†,‡^	0.34 ± 0.02^†,‡^	0.15 ± 0.02^†,‡^	6.68 ± 0.06^†,‡^

Data are expressed as mean ± SD (n = 6).

One-way ANOVA Tukey *post hoc*:

^†^p < 0.05 compared with CCl_4_.

^‡^p < 0.05 compared with vehicle control.

^§^p < 0.05 compared with silymarin.

Dosage: Olive oil: 2.4 ml/kg BW. CCl_4_ + Olive oil (1:4) Sol.: 3 ml/kg BW. NPs: 1 ml (OD_430_ 0.5)/kg BW (L) 1.5 ml (OD_430_ 0.5)/kg BW (M) 2 ml (OD_430_ 0.5)/kg BW (H). Silymarin: 1.5 ml/kg BW. Citrate: 750 µl/kg BW.

ANOVA: Analysis of variance; NP: Nanoparticle.

**Table 2.** **Summary of hematology parameters studied across the groups.**

**Parameters**	**Groups**					
	**I Control**	**II CCl_4_**	**III NP**	**V CCl_4_ + NP**	**VII CCl_4_ + citrate**	**VIII CCl_4_ + silymarin**
Hb (g/dl)	11.9 ± 1.2	8.8 ± 0.6^†^	12.3 ± 1.1^‡^	12.6 ± 0.7^‡^	11.8 ± 1.3^‡^	11.4 ± 1.1^‡^
RBC (×10^6^/μl)	10.8 ± 0.7	9.0 ± 0.4^†^	10.2 ± 0.8^‡^	11.1 ± 0.2^‡^	10.6 ± 0.4^‡^	10.5 ± 0.2^‡^
RT (%)	2.8 ± 0.2	4.9 ± 0.5^†^	3.0 ± 0.1^‡^	3.4 ± 0.4^‡^	3.2 ± 0.3^‡^	3.3 ± 0.3^‡^
HCT (%)	34.8 ± 2.5	30.0 ± 2.2	35.2 ± 2.4	35.2 ± 3.6	31.2 ± 2.5	33.9 ± 2.1
MCV (fl)	37.0 ± 2.9	32.4 ± 3.1	34.6 ± 3.2	36.8 ± 3.8	37.1 ± 3.2	36.8 ± 2.9
MCH (pg)	21.1 ± 2.1	20.2 ± 1.7	21.8 ± 1.5	21.5 ± 1.4	22.0 ± 1.8	21.7 ± 1.7
MCHC (g/dl)	41.4 ± 3.2	31.6 ± 2.1^†^	40.6 ± 3.8^‡^	40.2 ± 3.8^‡^	39.6 ± 4.3^‡^	34.9 ± 3.2
Platelets (×10^3^/μl)	6.6 ± 0.7	6.1 ± 0.6	6.6 ± 0.4	5.9 ± 0.6	5.8 ± 0.3	5.7 ± 0.5^†^
WBC (×10^5^/μl)	8.8 ± 0.4	13.0 ± 0.8^†^	8.6 ± 0.3^‡^	7.1 ± 0.5^‡^	6.4 ± 0.4^‡^	8.2 ± 0.3^‡^
L	76 ± 5.1	78 ± 6.3	74 ± 5.8	72 ± 6.2	76 ± 5.8	75 ± 5.1
N	25 ± 2.3	20 ± 1.8^†^	24 ± 1.6^‡^	21 ± 1.5	19 ± 2.1	24 ± 1.8^‡^

Data are expressed as mean ± SD (n = 6).

One-way ANOVA Tukey *post hoc*:

^†^p < 0.05 compared with vehicle control.

^‡^p < 0.05 compared with CCl_4_.

^§^p < 0.05 compared with silymarin.

Dosage:- Olive Oil: 2.4 ml/kg BW. CCl_4_ + Olive Oil (1:4) Sol.: 3ml/kg BW. NPs: 1.5 ml (OD_430_ 0.5)/kg BW. Silymarin: 1.5 ml/kg BW. Citrate: 750 µl/kg BW.

ANOVA: Analysis of variance; Hb: Hemoglobin; HCT: Hematocrit; L: Lymphocyte; MCH: Mean corpuscular hemoglobin; MCHC: Mean corpuscular hemoglobin concentration; MCV: Mean corpuscular volume; N: Neutrophil; RBC: Total red blood corpuscle; Rt: Reticulocyte; WBC: Total white blood corpuscle.

**Executive summary**

**Background**

Fibrosis, associated cirrhosis, and late stage of progressive scarring in chronic liver disease if untreated may lead to development of cancer, morbidity and even mortality as current therapeutics for management of these diseases are insufficient, poorly effective, time consuming and contains severe side effects.
As free radicals and oxidative stress play an important role in both onset and progression of liver fibrosis, NPs with antioxidant properties can be used as therapeutic agents. However, uses of inorganic NPs in treatment of chronic diseases are sparse in literature with problems in route of administration.

**Outcomes of the study**

In this study, the authors demonstrate that orally treated citrate functionalized Mn_3_O_4_ NPs can restore normal liver structure and function via antioxidant activity in a specific, nontoxic way compared with conventional drugs in mice model.

**Methods**

The *in vitro* pH dependent antioxidant activity of NPs has been shown and the detailed mechanism involved has been described.
*In vivo* preclinical studies of Mn_3_O_4_ NP as a therapeutic agent against liver fibrosis and associated disorders were done using Swiss albino mice as a model organism.
The efficiency of Mn_3_O_4_ NP in treatment of fibrosis in mice is ensured by biochemical tests and histopathological studies, with probable mechanistic insight.

**Conclusion & future perspective**

Our results confirmed that Mn-based NPs are nontoxic, biocompatible and effective probes against hepatic fibrosis, cirrhosis and associated disorders.
To the best of our knowledge, this is the first study that demonstrates direct oral treatment of an inorganic NP (i.e., C-Mn_3_O_4_ NPs) without any delivery system can efficiently reduce chronic hepatotoxicity and liver fibrosis.
This study may pave a new way for faster, safer and efficient therapeutic treatment of chronic liver diseases. This approach may be applied for future nanomedicine applications.
